# Recurring RNA structural motifs underlie the mechanics of L1 stalk movement

**DOI:** 10.1038/ncomms14285

**Published:** 2017-02-08

**Authors:** Srividya Mohan, Harry F Noller

**Affiliations:** 1 Center for Molecular Biology of RNA and Department of Molecular, Cell and Developmental Biology, University of California at Santa Cruz, Santa Cruz, California 95064, USA

## Abstract

The L1 stalk of the large ribosomal subunit undergoes large-scale movements coupled to the translocation of deacylated tRNA during protein synthesis. We use quantitative comparative structural analysis to localize the origins of L1 stalk movement and to understand its dynamic interactions with tRNA and other structural elements of the ribosome. Besides its stacking interactions with the tRNA elbow, stalk movement is directly linked to intersubunit rotation, rotation of the 30S head domain and contact of the acceptor arm of deacylated tRNA with helix 68 of 23S rRNA. Movement originates from pivoting at stacked non-canonical base pairs in a Family A three-way junction and bending in an internal G-U-rich zone. Use of these same motifs as hinge points to enable such dynamic events as rotation of the 30S subunit head domain and in flexing of the anticodon arm of tRNA suggests that they represent general strategies for movement of functional RNAs.

## Abstract (web summary)

Translocation of the tRNA on the ribosome is associated with large-scale molecular movements of the ribosomal L1 stalk. Here the authors identify the key determinants that allow these dramatic movements, and suggest they represent general strategies used to enable large-scale motions in functional RNAs.

It has become clear from extensive studies using a wide range of biochemical, biophysical and structural approaches that the ribosome is a complex molecular machine, with many moving parts. By far the most dramatic dynamic events are associated with the step of the elongation phase of protein synthesis known as translocation^1^. During translocation, the tRNAs are moved from the A-site to the P site to the E site. These movements of tRNA must be precisely coupled to movements of the mRNA by a single codon, in order to preserve the translational reading frame. Orchestration of this complex and biologically crucial process depends on coordinated movement of dynamic structural elements of the ribosome, which appear to guide and escort the tRNAs and mRNA between their binding sites^2^,^3^,^4^,^5^,^6^. Most extensively studied are the rotational movements of the 30S subunit^5^,^7^,^8^,^9^ and its head domain^10^,^11^,^12^, which play major roles in the movement of the acceptor ends of the tRNAs into their hybrid states, and translocation of the mRNA and anticodon ends of the tRNA, respectively.

Here we examine another prominent dynamic feature of the ribosome, the L1 stalk, which undergoes the largest-scale structural movements that have so far been observed for the ribosome, comparable to the largest excursions made by motor proteins^13^, during its participation in the translocation process^14^,^15^,^16^,^17^,^18^,^19^,^20^,^21^. The structure of the L1 stalk from a bacterial ribosome is shown in Fig. 1, along with its location in the 50S ribosomal subunit and the secondary structure of its rRNA moiety. Structural and single-molecule fluorescence resonance energy transfer (FRET) studies have shown that the L1 stalk moves through at least three different positions, corresponding to three well-characterized intermediate states of the translocation cycle^6^,^18^,^19^,^20^,^21^,^22^,^23^, representative structures of which are shown in Fig. 2. Tensor analysis^18^ of cryo-EM and X-ray structures, comparison of an ensemble of cryo-EM structures^24^ and single-molecule FRET studies^21^,^23^ have indicated correlated movement between L1 stalk movement, intersubunit rotation and tRNA translocation.

In classical-state ribosomes containing a vacant E site, the L1 stalk is found in an open conformation, with its head domain oriented away from the core of the ribosome (Figs 1 and 2a). Following peptide bond formation, the peptidyl-tRNA occupying the ribosomal P site becomes deacylated; its acceptor stem then moves into the E site of the 50S subunit, forming the P/E hybrid state, accompanied by inward movement of the L1 stalk, which establishes contact between its head domain and the elbow of the tRNA^16^,^19^,^25^,^26^,^27^ (Fig. 2b). The stalk then maintains contact with the tRNA elbow as it follows the progressive movement of the deacylated tRNA through the pe/E chimeric hybrid state^2^,^11^,^12^,^28^ (Fig. 2c) into the classical E/E state^29^,^30^,^31^,^32^ (Fig. 2d). Before this work, the position of the L1 stalk in the chimeric hybrid state had not been characterized as a unique intermediate state. Release of the deacylated tRNA from the ribosome presumably occurs on transition to the open state.

In this study, we address several key questions. First, what is the functional role (or roles) of L1 stalk movement? Second, how are its movements coordinated with the numerous other dynamic events associated with ribosomal translocation? Third, what is the structural basis of L1 stalk movement—that is, where exactly does movement originate, and which structural features are responsible? And finally, are there common structural principles underlying the molecular movements observed for different functional RNAs?

Our approach uses quantitative comparative structural analysis of an extensive database of 32 high- and medium-resolution X-ray and cryo-EM structures of ribosome complexes captured in intermediate states of translocation. As a frame of reference, we use a minimal model for translocation based on the four well-characterized functional states of the ribosome described above. Our findings point to a role for the L1 stalk in helping to coordinate movements of tRNA with movements of dynamic elements of the ribosome and in creating a possible checkpoint for the translocation process. Analysis of the trajectories of stalk movement between its different states shows that movement originates at the same RNA hinge points for transitions between each of the states. Most interestingly, the presence of similar structural motifs at the flexing points of other dynamic RNAs suggests that they represent a common set of strategies that are used for movement in many functional RNAs.

## Results

### L1 stalk dynamics and tRNA movement

We carried out a quantitative comparative structural analysis on a data set of 32 X-ray and cryo-EM structures of ribosome complexes from the Protein DataBank (PDB)^33^ in which the L1 stalk was well ordered, which includes both bacterial and eukaryotic ribosome complexes (Supplementary Table 1). We restricted our study to crystal structures with reported resolutions of 4 Å or better and cryo-EM structures with resolutions of 8 Å or better.

**Figure 1:**
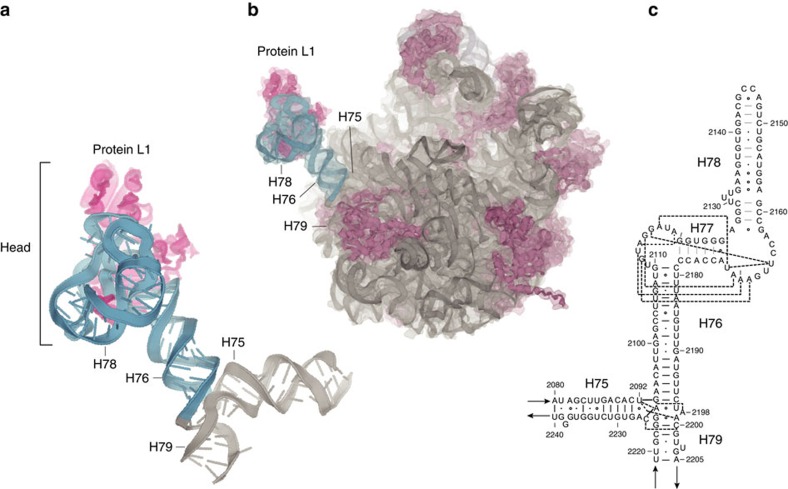
Structure and position of the L1 stalk in the 50S ribosomal subunit. (**a**) The L1 stalk comprises helices H76, H77 and H78 of 23S rRNA (blue) and protein L1 (magenta). It is connected to static helices H75 and H78 (grey). (**b**) Position of the L1 stalk in the *E. coli* 50S subunit in its orientation in the classical (open) state, in ribosomes containing a vacant E site (PDB ID 4GD2)^26^; the structure of protein L1, which was not modelled in 4GD2, has been docked based on its position relative to the L1 stalk RNA in the *T. thermophilus* structure (PDB ID 4V9K)^28^. (**c**) Secondary structure of the L1 stalk region of *E. coli* 23S rRNA. Tertiary interactions are indicated by dashed lines.

The structure of the L1 stalk begins with the long 23S rRNA helix H76, which is connected at its base to the body of the 50S subunit through helices H75 and H79; at its distal end, helices H77 and H78 are connected by tertiary interactions to create the compact fold of the head domain, which binds ribosomal protein L1 (Fig. 1). To identify the boundary between mobile atoms of the L1 stalk and the remainder of the 23S (or 28S) rRNA, we first performed 3D superimpositions of the static structural cores of their large-subunit rRNAs, as described in ‘Methods' section. This procedure distinguished the static H75 and H79 helices from the dynamic elements of H76 along with the more distal features of the stalk (Fig. 1a).

To quantify the magnitude and direction of L1 stalk movement, we applied the Euler–Rodrigues (E–R) method^34^ in which movement of a tethered rigid body between two conformational states is represented as a single rotational event about a calculated axis (E–R axis). The E–R axis for movement of the L1 stalk is nearly aligned to the helical axis of the fixed helix H75 (Supplementary Fig. 1), which connects orthogonally to H76 at the base of the stalk (Fig. 1). Localization of the E–R axis thus places the origin of stalk movement near the junction of H75 and H76.

**Figure 2:**
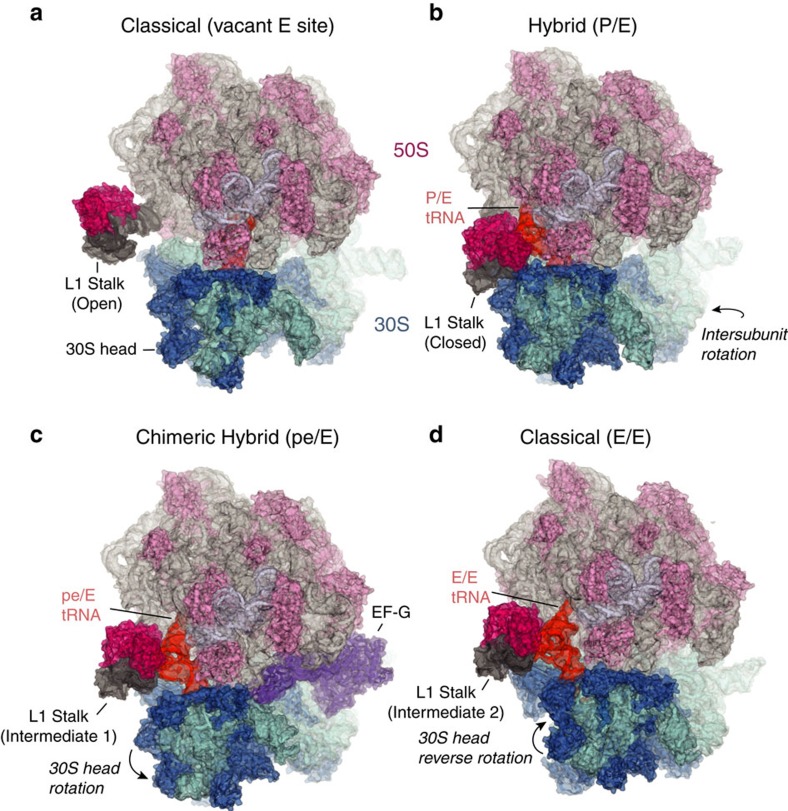
Positions of the L1 stalk in four different functional states of the ribosome. (**a**) Open (classical state; vacant E site; PDB ID: 4GD2)^26^; (**b**) Closed (hybrid P/E state; PDB ID: 4V9H)^16^; (**c**) Intermediate 1 (chimeric hybrid pe/E state; PDB ID: 4V9K)^36^; (**d**) Intermediate 2 (classical E/E state; PDB ID: 4V67)^58^ Components shown are: 23S rRNA moiety of the L1 stalk (dark grey); L1 protein (magenta, docked in (**a**) from Zhou *et al*.^28^); deacylated tRNA (orange); 16S rRNA (cyan); 23S rRNA (light grey); 5S rRNA (blue-grey); 30S proteins (dark blue); 50S proteins (light magenta). The head of the L1 stalk contacts the elbow of the deacylated tRNA as it moves from states (**b**–**d**), and the 30S subunit only in state (**b**). Directions of intersubunit and 30S head rotation are indicated.

**Figure 3:**
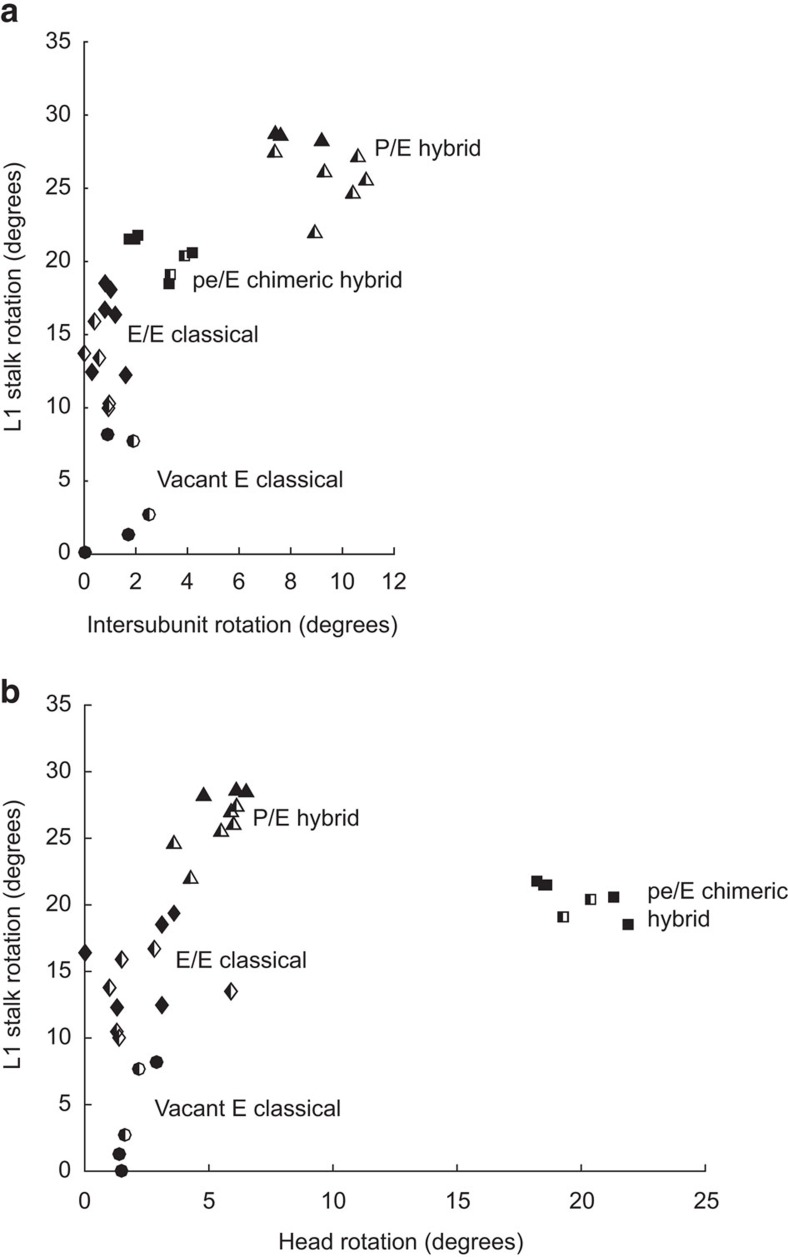
Rotation of the L1 stalk as a function of 30S subunit head and body rotation. (**a**) L1 stalk rotation versus 30S subunit body (intersubunit) rotation. (**b**) L1 stalk rotation versus 30S subunit head rotation. Rotation values were calculated using the E–R transform (Methods). Measured values cluster into four regions, corresponding to vacant classical (circles), E/E classical (diamonds), P/E hybrid (triangles) and pe/E chimeric hybrid (squares) functional states; filled symbols are from X-ray structures and half-filled symbols are from cryo-EM structures (Supplementary Table 1).

The magnitude of L1 stalk rotation values ranges from 0 to 30° (Table 1 and Supplementary Table 1) between its open conformation in the vacant classical-state ribosome (Fig. 2a) and its closed conformation in the hybrid-state ribosome containing a deacylated tRNA bound in the P/E state (Fig. 2b). Rotation values are strongly clustered for each binding state of the deacylated tRNA, and are correlated with the functional states of the ribosome and the magnitudes of the associated rotations of the head and body domains of the 30S subunit (Fig. 3). During formation of the P/E hybrid state, the stalk undergoes a large-scale inward rotation through a distance of up to 63 Å to its maximally rotated (closed) state, initiating contact between the head domain of the stalk and the elbow of the P/E tRNA (Fig. 2b). In this same transition, the adjacent surface of the head of the stalk contacts a complementary surface on the 30S subunit, as discussed below. Moreover, the major groove near the proximal end of H76 widens dramatically from 11 to 20 Å (as measured between P2093 and P2189), as H76 moves into its closest approach to helix H75 (Supplementary Fig. 2). The closed conformation is observed in hybrid-state ribosomes containing either a single tRNA bound in the P/E state^9^,^26^,^35^ or two tRNAs bound in the A/P and P/E states^5^,^27^.

When the tRNA next moves into its chimeric hybrid pe/E state^2^,^11^,^12^,^36^, the stalk moves outward by ∼10 Å to a rotation value of about 20°, a conformation that we call ‘intermediate 1', not previously classified as a distinct state of the L1 stalk (Fig. 2c). Movement of the tRNA from its pe/E chimeric hybrid state to the classical E/E state is accompanied by a further ∼8 Å outward movement of the L1 stalk to a rotational value of about 15°, while maintaining its contact with the tRNA elbow (Fig. 2d). We term this state ‘intermediate 2' (previously called ‘half-closed'^22^). Finally, outward rotation of the L1 stalk by an additional 11 Å into its open conformation (Fig. 2a) allows release of the deacylated tRNA from the ribosome.

**Figure 4:**
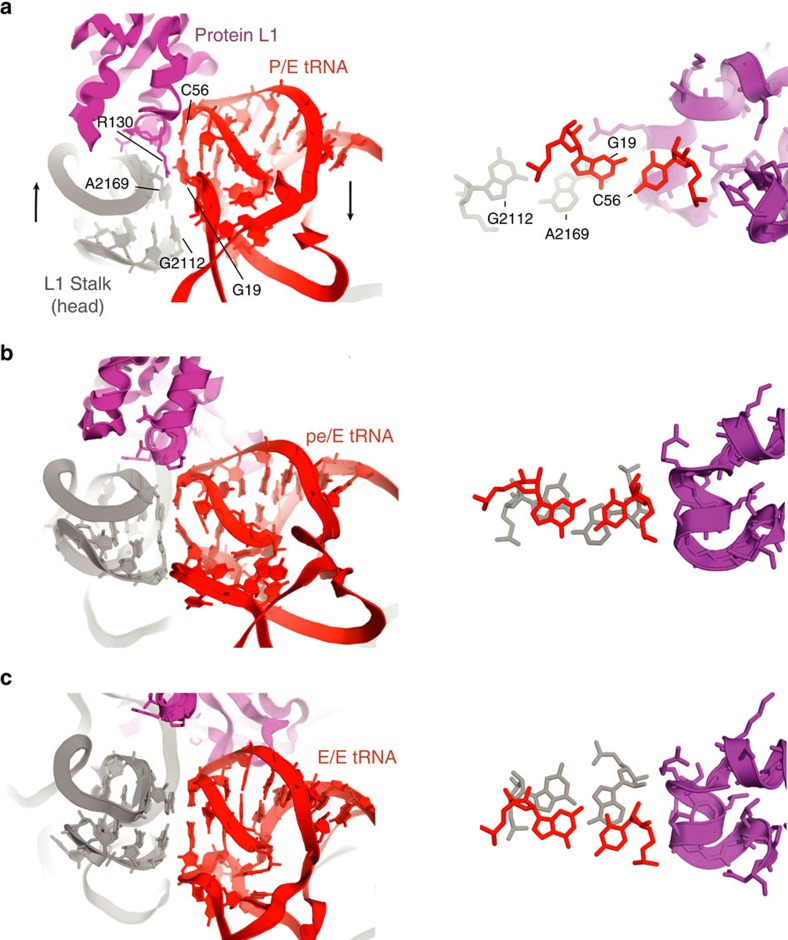
Stacking of rRNA elements of the head of the L1 stalk on the elbow of the deacylated tRNA. (**a**) Hybrid P/E state (PDB ID: 4V9H)^16^; (**b**) chimeric pe/E state (PDB ID: 4V9K)^36^; and (**c**) classical E/E state (PDB ID: 4V67)^58^, showing the 23S rRNA (grey) and L1 protein (magenta) components of the L1 stalk and the elbow of the deacylated tRNA (red). The extent of overlap between stacked bases is shown in the right-hand panels. (Missing domains of protein L1 in **c** were docked based on their positions relative to the L1 stalk RNA in 4V9K)^28^.

Contact between the head domain of the L1 stalk and the deacylated tRNA involves stacking of the non-canonical G2112–A2169 base pair of the stalk rRNA on the tertiary G19–C56 Watson–Crick pair on the elbow of the tRNA (Fig. 4), as observed in several X-ray crystal structures^16^,^19^,^26^,^28^. Although contact between the head of the L1 stalk and elbow of the deacylated tRNA is maintained throughout movement of the tRNA, the nature and stacking overlap of the contacting surfaces change during progression through the different states of translocation (Fig. 4). In the P/E hybrid state, contact is formed mainly between the G19–C56 tertiary Watson–Crick base pair in the tRNA elbow and helix α3 of protein L1, with minimal participation of the RNA moiety of the L1 stalk (Fig. 4a). In the pe/E chimeric hybrid state, the head of the stalk slides to maximize stacking overlap between the non-canonical G2112–A2169 tertiary base pair of the L1 stalk RNA and the G19–C56 pair, while interaction with protein L1 is greatly reduced (Fig. 4b). Finally, in the E/E classical state, backbone atoms of the tRNA D loop contact the L1 stalk at the junction between H76 and the head of the stalk (Supplementary Fig. 3), while stacking overlap between G2112–A2169 and G19–C56 is nearly eliminated as the tRNA approaches the end of its occupancy in the ribosome (Fig. 4c).

### The structural basis of L1 stalk movement

**Figure 5:**
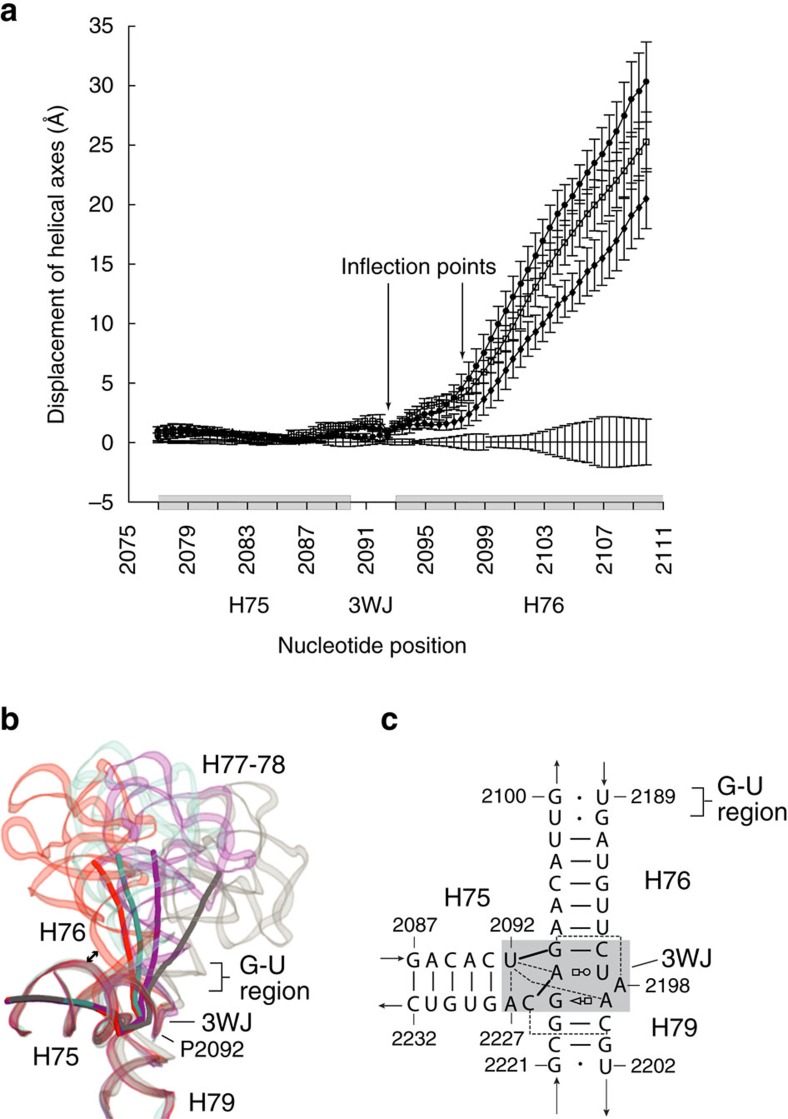
Localization of the origins of movement of the L1 stalk. (**a**) Deviation of the helical axis of 23S rRNA in the L1 stalk in the P/E hybrid (circles); pe/E chimeric (squares); and E/E classical (diamonds) rotated states, relative to the vacant classical state (baseline). Calculated average helical displacement for each group with error bars representing s.d. is shown. Inflection points are seen around positions 2092 and 2098. (**b**) Positions of the L1 stalk and its helical axis in the vacant classical (grey); P/E hybrid (red); pe/E chimeric hybrid (cyan); E/E classical (magenta) states, showing the positions of hinging in the three-way junction (3WJ) and bending in the G-U-rich region of H76. Closest approach (3.6 Å) of H76 in the L1 stalk to H75 is indicated by the arrow. (**c**) Secondary structure of 23S rRNA around the 3WJ (shaded). Non-canonical base pairs are represented by the symbolic nomenclature described by Leontis *et al*.^66^. Dotted lines represent single hydrogen bonds, which except between U2092 and A2227, are between non-coplanar residues.

We localized the origin of L1 stalk movement by calculating the deviation of the axes of the helical elements of the stalk relative to their positions in the classical state (Fig. 5a) (see ‘Methods' section). The first major inflection point occurs around position 2092, within the linker connecting H75–H76 (Fig. 5a,b); a second one is found around position 2098 within H76 (Fig. 5a). The deviation plots show that although H76 is displaced by different magnitudes in the different functional states of its ribosome complexes, the inflection points occur at the same positions for each transition (Fig. 5a). The inflection around position 2092 lies within the Family A three-way junction^37^ formed by helices H75, H76 and H79 (Fig. 5c), while the inflection around position 2098 coincides with the G-U-rich region within H76 (Fig. 1c), implicating both regions in rotation of the L1 stalk.

**Figure 6:**
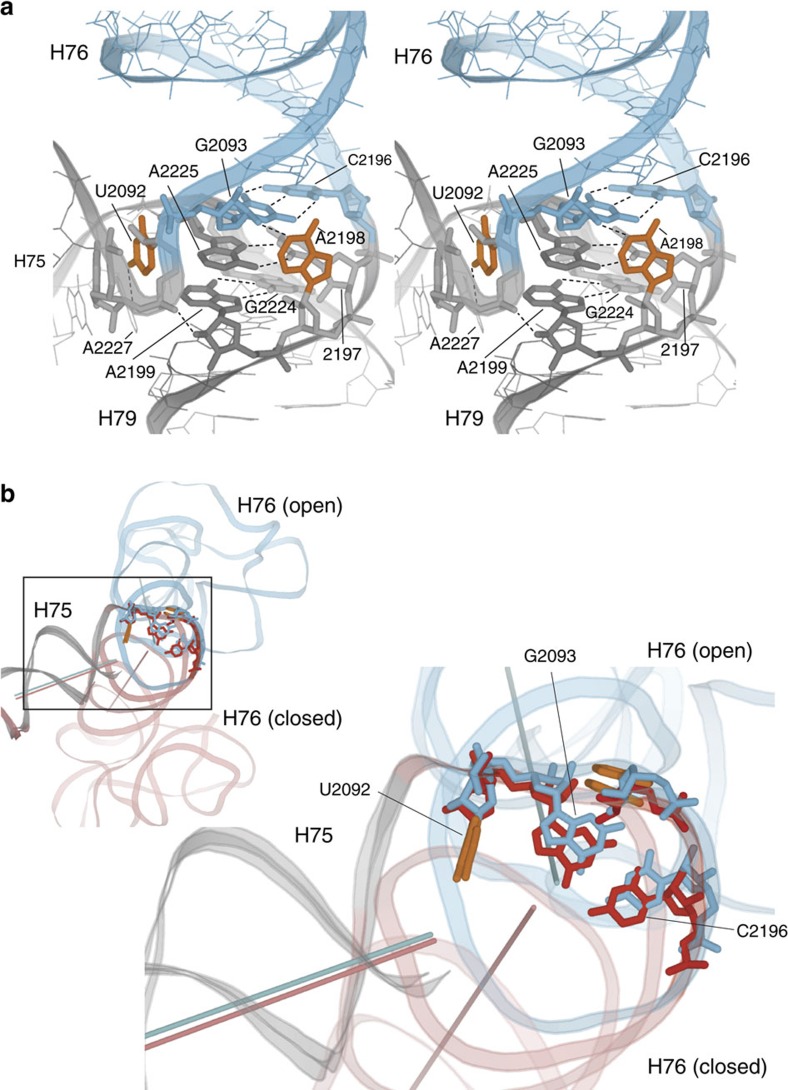
Structural interactions at the three-way junction. (**a**) Stereo view of the 3WJ showing coaxial stacking of H76 (blue) and H79 (grey) and orthogonal orientation of H75 (grey). U2092 and A2198 (orange) pack against opposite minor groove surfaces perpendicular to the coaxial helical axis at the core of the junction. A sharp bend is formed by nucleotides 2091–2093 in the linker joining H75 and H76. A series of stacked purines is found at the core of the junction, contained in the sheared A2199–G2224, Hoogsteen A2225–U2197 and Watson–Crick G2093–C2196 pairs. (**b**) View of the 3WJ from H79 showing the positions of the helical axes of H75 and H76 in the open (blue) and closed (red) states. Changes in the positions of nucleotides between the open and closed states of the L1 stalk show that the origin of movement of H76 lies within the internucleotide linkage between U2092 and G2093. (PDB IDs: open L1 stalk, 4GD2)^26^; closed, 4V90)^67^.

Helices H75 and H79, two of the three helices at the three-way junction, lie perpendicular to each other, but remain static during movement of the dynamic H76. U2092, at the first inflection point of the deviation plot, is positioned precisely at a sharp bend formed between nucleotides 2091–2093 in the linker between H75 and H76 (Fig. 6a). Although U2092 itself remains fixed, the very next position, G2093, is the first nucleotide to show clear mobility (Fig. 6b), localizing the pivot point for the L1 stalk to the sharp bend, within the 2092–2093 internucleotide linkage. Movement thus originates at the stacking interface between the Watson–Crick G2093–C2196 at the end of H76 and the Hoogsteen A2225–U2197 pair in the core of the three-way junction (Fig. 6a).

U2092 lies at the end of helix H75 but packs perpendicularly against the minor groove of H79 near the three-way junction (Fig. 6a), apparently helping to mutually restrain movement of helices H75 and H79. Similarly, in the linker joining the coaxial helices H76 and H79, base A2198 packs against the minor groove at the end of H76 (Fig. 6a). We suggest that this unusual perpendicular packing of bases 2092 and 2198 against opposite minor groove surfaces around the junction may help to constrain the direction and extent of motion of the L1 stalk through all its movements. Perpendicular packing of a base against the minor groove also occurs in the decoding site of the 30S subunit^38^, where G530 packs orthogonally against the minor groove of the codon-anticodon helix, contacting position 35 of the anticodon of the A-site tRNA.

**Figure 7:**
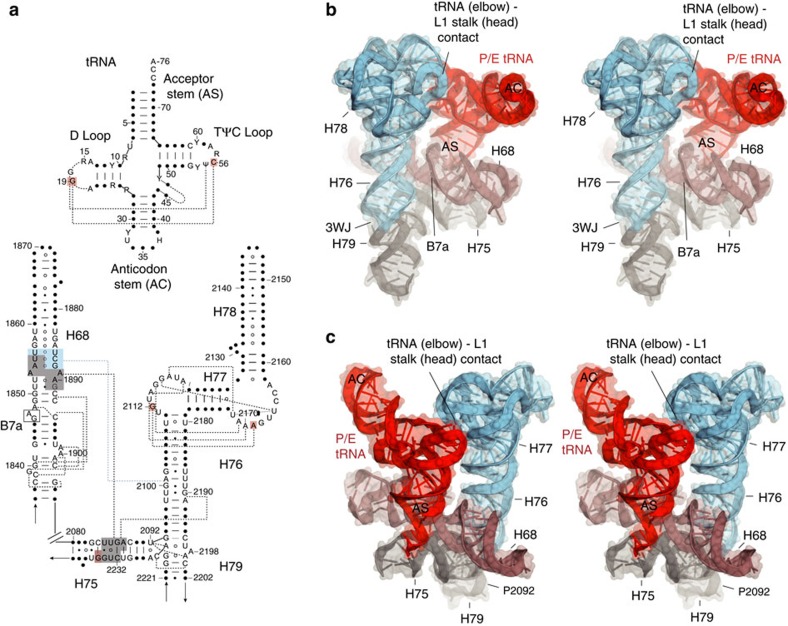
A transient network of minor groove interactions connects the tRNA with rRNA helices. (**a**) Secondary structure diagram of tRNA and elements of 23S rRNA showing minor-groove interactions between tRNA and rRNA (red shading); H68 and H76 (blue shading); and H68-H75 (grey shading). The point of contact between H68 and 16S rRNA to form intersubunit bridge B7a is indicated. Tertiary interactions are shown as dashed lines. (**b**,**c**) Two views showing the central role of H68 (brown) in its network of minor-groove packing interactions with H76 in the L1 stalk (blue), H75 (grey) and the acceptor arm of deacylated tRNA (red) in the P/E hybrid state (PDB ID: 4V90)^67^. The point of contact between H68 and 16S rRNA to form intersubunit bridge B7a is indicated in (**b**).

Although hinging at the three-way junction dominates all L1 stalk transitions (Fig. 5a,b), a secondary inflection occurs around U2098, due to bending of H76 in the region containing multiple G-U wobble pairs^15^,^19^,^39^ (Fig. 5a,c, Supplementary Fig. S4). Flexing within this region of H76 is most pronounced in the hybrid-state and chimeric hybrid-state conformations (Fig. 5a), allowing the L1 stalk to maintain contact with the elbow of the translocating tRNA through its largest excursions. A G-U pair at a position corresponding to the G2100–U2189 pair is conserved in bacteria, eukarya and archaea^40^; interestingly, minor groove contact between H76 and H68 (discussed below) is centred on this same conserved G-U pair (Fig. 7, Supplementary Fig. S5). In general, H76 is seen to contain multiple G-U pairs, base–base mismatches or (in eukarya) single-base bulges, any of which would predispose it to bending, as seen in the structures that have been determined so far. Thus, the overall movement of the L1 stalk results from combined pivoting within the three-way junction and bending within the G-U-rich section of helix H76.

### A complex network of minor groove interactions

The 23S rRNA helix H68 is wedged in a complex network of minor groove interactions involving H75, H76 and the tRNA (Fig. 7, Supplementary Fig. 5, Supplementary Movie 2). H68 maintains constant contact with the static H75 through four conserved adenosines (A1853, A1854, A1889 and A1890) to form a series of consecutive stacked Type I and Type II A-minor interactions^41^(Supplementary Fig. 5). This site of contact on H68 is flanked by minor groove interacts with two dynamic elements—helix H76 of the L1 stalk and the acceptor stem of the deacylated tRNA (Fig. 7b,c). The minor groove of the acceptor arm of the deacylated tRNA contacts H68 as it enters the 50S E site (positions 1850–1852, 1891–1893 on H68). Molecular dynamics studies have predicted that U1851 flips out toward the P/E tRNA during translocation between the P/E and E/E states^42^, although such a conformation has not been observed in the structures of any intermediates so far. As the L1 stalk moves to contact the elbow of the translocating tRNA, it also forms minor groove interactions with H68. Interestingly, it is the minor groove surface of the G-U-rich bending region of H76 that contacts the minor groove surface of H68 via an extensive interface in all but the open L1 stalk position (Fig. 5b). Thus, H68 may coordinate multiple dynamic events around the 50S E site, including limiting the range of stalk motion at the three-way junction.

### Contact between the L1 stalk and the 30S subunit

Uniquely in the P/E hybrid-state ribosome, movement of the L1 stalk creates contact between complementary surfaces of its head domain and the 30S subunit (Fig. 2b, Supplementary Fig. 6; Supplementary Movie 1), a transient intersubunit bridge we call B9. These contacts, which had gone unnoticed until recent low-resolution cryo-EM^24^ and FRET-based studies^23^, are formed between G2141 (H78) of 23S rRNA with protein S11 in the 30S body domain; positions G2116 (H77) and G2148 (H78) with S7 in the 30S head domain; and protein L1 with protein S13 in the 30S head domain. The interaction involving G2116 accounts for its protection from kethoxal modification in ribosomes containing P/E state tRNA^25^. The potential functional implications of this contact are discussed below.

### Eukaryotic L1 stalk structure and dynamics

Although there are fewer available eukaryotic structures, the mechanics of L1 stalk movement are likely to be very similar (Supplementary Figs S7 and S8). The length of the stalk and the position and topology of the three-way junction at the base of the L1 stalk are essentially identical to their bacterial counterparts (Supplementary Fig. S7), as seen in the eukaryotic structures containing a fully modelled L1 stalk^43^,^44^,^45^,^46^,^47^,^48^. Even though position U2092 within the three-way junction is replaced with guanine in mammalian ribosomes^43^,^44^,^45^, it interacts similarly with position A2227 (also an adenine in eukaryotes), by forming a single hydrogen bond; U at position 2092 is conserved in yeast^49^,^50^ and *Plasmodium*^51^. A single G-U wobble pair is found within the G-U-rich region, corresponding to the conserved G2100–U2189 pair in the bacterial structures, which in the P/E hybrid state^44^, participates in minor groove contacts with the bacterial equivalent of H68. In an earlier 11.7 Å cryo-EM structure of the yeast 80S·eEF2·sordarin complex, Spahn *et al*.^15^ localized hinge points close to the three-way junction and at a bulged base near the G-U-rich region, in close agreement with our findings.

There are a handful of notable differences between the eukaryotic and prokaryotic L1 stalks. The head domain of the eukaryotic L1 stalk lacks the structural equivalent of bacterial helix H78. Nevertheless, we note that in recent high-resolution cryo-EM structures of the mammalian ribosome containing tRNA bound in the A/P and P/E hybrid states^44^, rRNA elements of the head of the L1 stalk establish contact with the head of the small subunit through molecular interactions with protein S25 (Supplementary Fig. 7b). In this particular structure, the L1 stalk does not contact the body of the 40S subunit, unlike what is seen in hybrid states bacterial structures. Also unique to eukaryotic ribosomes is an extended open conformation of the stalk in the presence of IRES elements^46^,^47^,^48^ (Supplementary Fig. 8). Comparison of the limited number of eukaryotic L1 stalk structures in the extended open, closed and intermediate two positions shows pronounced hinging centred on the three-way junction at the base of the stalk and flexibility around the position of the conserved G-U pair (Supplementary Fig. 8).

## Discussion

In this study, quantitative structural analysis of 32 X-ray and cryo-EM structures of ribosome complexes containing well-ordered L1 stalks shows that it occupies at least four distinct positions, corresponding to four well-characterized functional states of the ribosome (Table 1, Supplementary Table 1; Figs 2 and 3). During conformational transitions between these states, the head domain of the L1 stalk moves through a distance of more than 60 Å by hinging of helix H76 at the Family A three-way junction formed by helices H75, H76 and H79 (ref. 37) together with bending at the G-U-rich section in the middle of helix H76. Hinging at the three-way junction is localized to a point within the U2092–G2093 internucleotide linkage. As the stalk moves into its closed (P/E hybrid) state, its head domain contacts the elbow of the deacylated P/E tRNA, and maintains this contact during translocation of the tRNA through its final classical E/E binding state. This interaction is preserved by bending of H76 in its G-U-rich region at its point of contact with helix H68.

In the transition from the open to the closed state, a network of minor-groove interactions is formed around H68 of 23S rRNA. Inward movement of the stalk creates a new contact between the minor groove surface of the G-U-rich region of H76 and the minor groove of H68 at positions 1856/1886–1888, immediately adjacent to the fixed minor groove interaction between H75 and H68 (Fig. 7). Movement of the deacylated tRNA into the P/E state creates yet another minor-groove contact between the backbone atoms of H68 at positions 1850–1852 and 1892–1893 with positions 1–4 and 70–72 at the acceptor end of tRNA (Fig. 7; Supplementary Movie 2), explaining why methylation of ribose 71 of tRNA causes inhibition of translocation^52^. In a further interaction formed by H68, positions 1846–1848 contact helix h23 of 16S rRNA in the 30S subunit around position A702 to form intersubunit bridge B7a^53^. Thus, helix H68 is implicated in coordinating multiple events involving movements of the L1 stalk, the acceptor stem of tRNA and the 30S subunit during translocation.

A critical aspect of RNA dynamics is that the range of motion of a dynamic element needs to be constrained, reducing the degrees of freedom in its trajectory to establish the precision of an associated functional process. Examination of contacts formed around the sites of flexing of the L1 stalk reveals several constraints on the range of L1 stalk movement. These include the unusual perpendicular packing of U2092 and A2198 against the minor groove surfaces surrounding the hinge point at the three-way junction (Fig. 6a) and the minor-groove interactions between H76 and H68 flanking the G-U-rich bending region (Supplementary Fig. 4).

Interestingly, the head of the L1 stalk forms an extensive interaction with the 30S subunit uniquely in the hybrid (closed) state, through contact between complementary surfaces (Fig. 2b, Supplementary Fig. S6). Helices H77, H78 and protein L1 in the head of the stalk contact proteins S7 and S13 in the 30S subunit head domain and S11 in the 30S body domain, thus forming a transient intersubunit bridge (bridge B9). Formation of this bridge uniquely in P/E hybrid ribosome structures suggests that it may play a special role in the translocation pathway. Since it occurs immediately preceding the rate-limiting step of large-scale rotation of the 30S subunit head domain^2^,^12^,^28^,^54^, this bridge must be disrupted in order for the ribosome to undergo the rate-limiting transition from the hybrid state to the chimeric hybrid state. As 30S head rotation is sterically blocked by contact with the L1 stalk, establishment of this bridge may thus serve as a checkpoint to ensure that the acceptor end of an authentic deacylated tRNA is secured in the 50S E site before movement of the mRNA and anticodon ends of the tRNAs in the 30S subunit.

Our findings point to some emerging principles of RNA functional dynamics. Together with our previous analysis of the mechanism of rotation of the 30S subunit head domain^34^ and earlier observations on flexing of tRNA^2^,^55^,^56^,^57^,^58^, the mechanisms underlying L1 stalk movement begin to point to a common set of strategies that enable the functional dynamics of RNA, which include (1) hinging at Family A 3-way junctions; (2) bending at G-U-rich and other weak regions of RNA helices; (3) flexing at junctions between coaxial helices terminating in purine–purine and other non-canonical base pairs; and (4) stacking of bases in RNA tertiary folds on the tRNA elbow.

The possible role of Family A three-way junctions in RNA dynamics has been suggested previously based on comparative structural analysis^37^, transient electric birefringence experiments^59^ and molecular dynamics simulations^39^,^60^. In addition to L1 stalk movement, rotation of the 30S subunit head domain during the second step of translocation has been shown to be based on hinging at a Family A three-way helical junction^34^.

A second theme is the observed flexibility of RNA helices in regions containing multiple G-U wobble pairs and other weak helical features. This is seen for the L1 stalk at the G-U-rich region in the middle of helix H76 (Fig. 1c). Previously, rotation of the 30S head domain was shown to utilize bending in helix h28 of 16S rRNA centred on the position of the bulged G926, which is flanked by two G-U pairs^34^.

A third strategy is the occurrence of non-canonical base pairs (most commonly purine–purine pairs) at the pivot point of hinging between coaxially stacked helices. In the case of the L1 stalk, hinging occurs at the junction of H76 and H79, where the non-canonical A2199–G2224 purine–purine pair at the base of H79 is stacked on the U2197–A2225 Hoogsteen pair at the base of H76 (Fig. 6). In tRNA, flexing occurs between the D and anticodon stems during aminoacyl-tRNA accommodation^55^,^56^,^57^ and translocation^2^,^5^,^26^,^28^. Here a purine–purine mismatch (typically G26-A44) is usually intercalated between the terminal Watson–Crick pairs of the two stems at the hinge point.

A fourth example, stacking and sliding between the surfaces of bases in tertiary folds appears to be a common strategy employed by RNAs that contact the elbow of tRNA. Stacking interactions similar to that observed between the tertiary base pairs of the elbow of the translocating tRNA and the head of the L1 stalk have been observed in RNaseP and in the T-box riboswitch^61^. The head of the L1 stalk, RNaseP and Stem 1 of the T-box riboswitch all fold into variations of the head-to-tail double T-loop module at their point of contact with the elbow of tRNA, suggesting that it is specifically adapted to maintaining contact with a dynamically flexing tRNA.

The finding that similar structural features are found repeatedly at the origins of movement in prominent examples of functionally important RNA dynamics suggests that they represent general principles for enabling movement in RNA. Another emerging idea is that the most crucial aspect of RNA movement may be restriction of the degrees of freedom of dynamic elements, as exemplified by movement of the L1 stalk; thermal energy alone may be sufficient to enable all of the movements involved in ribosome translocation, as has been seen, for example, in intersubunit rotation^62^. Finally, the fundamental role of RNA dynamics in the mechanism of protein synthesis raises a further argument supporting the choice of RNA as a founding molecule in the molecular origins of life. RNA is unique in its ability to carry out the three most critical functions necessary for the emergence of life: storage and replication of genetic information, catalysis of biological reactions and large-scale molecular movement.

## Methods

### Comparative structure data set

Our quantitative comparative structural analysis is based on a data set of 32 X-ray and cryo-EM structures of ribosome complexes from the PDB^33^ that contain complete L1 stalks. We restricted the data set to crystal structures with reported resolutions of 4 Å or better and cryo-EM structures with resolutions of 8 Å or better. Within this cut-off, any structures in which the L1 stalk appeared significantly disordered or deformed were eliminated. The majority of the data set consists of structures derived from bacterial ribosomes from *E. coli* or *T. thermophilus* and eukaryotic ribosomes from yeast and *Plasmodium* (Supplementary Table S1).

### Structural superimpositions

To identify the boundary between mobile atoms of the L1 stalk and the remainder of the 23S (or 28S) rRNA, we used PyMol^63^ to perform 3D superimpositions on the static core atoms of the rRNA whose structural equivalents are conserved across all structures used in this study. The core residues (listed below) were determined by comparison of atomic coordinates of the rRNA sugar-phosphate backbone. Atoms with <0.8 Å root mean square deviation (RMSD) were classified as static regions. We observe that the atoms of the helices H76, H77 and H78 are mobile across the 32 structures, but helices H75 and H79, which form a three-way junction with H76, remain static across all structures. Core residue positions used for superimposition are:

Bacterial 23S rRNA: 173–269, 292–344, 372–542, 552–626, 657–926, 938–1053, 1108–1373, 1375–1412, 1425–1478, 1552–1579, 1587–1718, 1744–1859, 1882–1906, 1930–2088, 2227–2787.

Yeast and *Plasmodium* 28S rRNA: 16–114, 180–234, 270–433, 626–704, 788–1062, 1109–1228, 1282–1554, 1583–1622, 1653–1705,1780–1807, 1819–1948, 2101–2219, 2225–2249, 2273–2430, 2596–3150.

### Alternate conformations of the L1 stalk

We calculated the magnitude of L1 stalk rotation using the E–R formula (see below) for each ribosome, relative to the position of the stalk in the X-ray structure of a classical-state *E. coli* ribosome complex containing a vacant E site (the open conformation) as a reference structure^26^ (PDB ID: 4V9D). Four distinct L1 stalk positions, correlating with four functional states of the ribosome, are grouped as open (classical ribosome, vacant E site), closed (P/E hybrid ribosomes, P/E tRNA), intermediate 1 (chimeric hybrid ribosomes, pe/E chimeric tRNA) and intermediate 2 (classical ribosomes, classical E/E tRNA).

### Calculating the magnitude and direction of domain movement

We applied the E–R method as described in ref. 24 to calculate the magnitude and direction of stalk movement in each ribosome complex, using the Pymol plug-in created for this purpose. The plug-in can be downloaded at http://rna.ucsc.edu/rnacenter/erodaxis.py.

The E–R method describes any movement of a mobile domain as a simple rotational event about a single calculated axis (the E–R axis), with respect to a reference state. For a rigid body rotational event, applying the E–R formula generates an axis and a corresponding angle of rotation for the mobile domains. The structures being compared must be aligned on their static domains. Pymol generates a composite 4 × 4 transformation matrix containing a 3 × 3 the rotation (R) matrix and 3 × 1 translation (T) matrix, for this alignment. The angle (Θ) of rotation is derived from diagonal elements of the rotation matrix as:


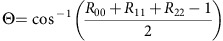


The direction of the axis is calculated as:


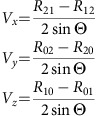


The origin of the axis can be described by 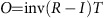, where *I* is a 3 × 3 identity matrix.

For purposes of quantifying stalk movement, the mobile domain is defined as helix H76 (residues 2093–2109, 2181–2196), while Helix H75 (residues 2083–2090, 2229–2236) is the static domain.

### Calculation of helical axes and axis deviations

Positions of the helical axes for different L1 stalks were determined using Curves+^64^, and grouped according to the functional states of the ribosome complexes and tRNA binding states. Deviation of coordinate positions along the helical axes relative to the positions for the average helical axis for the L1 stalk in the open position were calculated for each structure using Matlab^65^.

### Data availability

All data generated or analysed during this study are included in this published article and its Supplementary Information files, and are available from the corresponding author upon request.

## Additional information

**How to cite this article:** Mohan, S. *et al*. Recurring RNA structural motifs underlie the mechanics of L1 stalk movement. *Nat. Commun.* **8,** 14285 doi: 10.1038/ncomms14285 (2017).

**Publisher's note**: Springer Nature remains neutral with regard to jurisdictional claims in published maps and institutional affiliations.

## Supplementary Material

Supplementary Information Supplementary Figures, Supplementary Tables and Supplementary References

Supplementary Movie 1 The position of the L1 stalk correlates with the functional state of the ribosome. The position of the L1 stalk correlates with the functional state of the ribosome, which in turn, is closely correlated to the four binding states of the translocating tRNA from the P site to the E site. The L1 stalk occupies a distinct position in each state. Interpolations of the structural transitions through the four functional states were rendered using PyMol28 (PDB IDs - classical state with vacant E site, 4GD21; P/E hybrid state, 4V9023; chimeric pe/E state, 4V9L18 and classical state, with E/E tRNA, 4V6F7.)

Supplementary Movie 2 Network of minor-groove interactions around the 50S E site. Network of minor-groove interactions around the 50S E site. The 23S rRNA helix H68 is at the center of a complex network of minor groove interactions. H68 interacts with the static H75 at all times; however, this minor groove surface of H68 is flanked by two dynamic minor-groove interactions with the acceptor arm of the deacylated tRNA and H76 of the L1 stalk. H68 is also a point of contact with the 30S subunit though intersubunit bridge B7a. Interpolations of the structural transitions through the four functional states were rendered using PyMol28 (PDB IDs - classical state with vacant E site, 4GD21; P/E hybrid state, 4V9023; chimeric pe/E state, 4V9L18 and classical state, with E/E tRNA, 4V6F7.)

## Figures and Tables

**Table 1:** Correlation between L1 stalk position and ribosome functional state*.

**L1 stalk Position**	**Ribosome functional state**	**E site tRNA binding state**	**Contact with tRNA elbow**	Average L1 stalk rotation* **(°)**	Average L1 stalk displacement*† **(Å)**	**30S body rotation**‡ **(°)**	**30S head rotation**§ **(°)**	**N**||
Open	Classical(Pre-translocation)	Vacant	−	4.0±3.8	12.0±8.7	1.4±0.9	1.9±0.6	5
Closed	Hybrid	P/E	+	26.5±2.2	51.1±7.4	9.1±1.4	5.5±0.9	9
Intermediate 1	Chimeric hybrid	pe/E	+	20.4±1.2	42.2±1.2	3.0±1.0	19.7±1.5	7
Intermediate 2	Classical (Post-translocation)	E/E	+	14.4±2.9	31.9±6.4	0.8±0.5	2.2±1.6	11

^*^The values for L1 stalk rotation and displacement were measured relative to the position of the stalk in its most open conformation, from the structure of an *E. coli* 70S ribosome complex containing a single tRNA bound in the P/P state, defined as 0 (degrees) rotation and 0 (Angstroms) displacement PDB ID: 4GD2 (ref. 26).

^†^Measured between position P2127 in bacteria (P2469 in yeast) at the top of the L1 stalk head domain in each structure (Supplementary Table 1), relative to its position in the open state reference structure (4GD2). (See ‘Methods' section).

^‡^30S subunit body rotation (or intersubunit rotation), calculated using the E–R method between 16S rRNA body domains, relative to 4GD2 as the reference state.

^§^30S subunit head rotation, calculated using the E–R method, as described in Mohan *et al*.^34^.

^||^Number of structures used in the analysis, from X-ray and cryo-EM studies, including eukaryotic ribosomes. For a complete list of structures used, see Supplementary Table 1.
