# Supplementary material for: Recurring RNA structural motifs underlie the mechanics of L1 stalk movement
Source: Nat Commun. 2017 Feb 8;8:14285. doi: 10.1038/ncomms14285 (PMC5309774; doi:10.1038/ncomms14285)
Supplement: Supplementary Information — Supplementary Figures, Supplementary Tables and Supplementary References [file ncomms14285-s1.pdf]

Supplementary Table 1: Magnitude of rotation and displacement of the L1 stalk in different functional states of the ribosome

No.	References	PDB ID[a]	L1 Stalk Rotation[b] (°)	L1 Displace-ment[c] (Å)	30S BR[d] (°)	30S HR[e] (°)	tRNA Binding states	L1 stalk Position[f]	Ribosome State[g]	Ligands[h]	Sp[i]	Res[j] (Å)
1	Dunkle J., et al. (2011)[1]	4GD2	0	0	0	1.5	P	Open	Classical, PRE	-	Eco	3.0
2	Wasserman , MR., et al. (2015)[2]	4WOI	1.3	1.9	1.7	1.4	P	Open	Classical, PRE	Par	Eco	3.0
3	Sun, M., et al. (2015)[3]	3JBN*,	2.7	-	2.5	1.6	P	Open	Classical, PRE	-	Pfa	4.7
4	Zhang J, et al.(2016)	3JBU*	7.7	17.6	1.9	2.2	P	Open	Classical, PRE	SecM	Eco	3.3
5	Seefeldt, AC., et al. (2016)[4]	5F8K	8.2	16.6	0.9	2.9	P	Open	Classical, PRE	Bac7	Tth	2.8
6	Khatter, K. (2015)[5]	4UG0*	10.0	23.2	1.2	1.5	E	Intermediate 2	Classical, POST		Hsa	3.6
7	Behrmann E. et al (2015)[6]	5AJ0*	10.5	23.9	1.2	1.4	P, E	Intermediate 2	Classical, POST		Hsa , Yps	3.5
8	Jenner, LB, et al. (2010)[7]	4V6F	12.3	28.3	1.6	1.3	A, P, E	Intermediate 2	Classical, POST	-	Tth	3.1
9	Korostelev, A, et al. (2008)[8]	4V67	12.5	31.7	0.3	3.1	P, E	Intermediate 2	Classical, POST	RF-2	Tth	3.0
10	Sun, M., et al. (2015)[3]	3JBP*	13.5	-	0.6	5.9	E	Intermediate 2	Classical, POST	-	Pfa	6.7
11	Li, W, et al. (2015)[9]	3J9Z*	13.8	29.9	0	1.0	P, E	Intermediate 2	Classical, POST	EF-G.GTP	Tth	3.6
12	Zhang, D., et al. (2016)[10]	3JCD*	15.9	33.5	0.4	1.5	P, E	Intermediate 2	Classical, POST	EF4	Eco	3.7
13	Selmer, M, et al. (2006)[11]	4V51	16.4	28.8	1.2	0	A, P, E	Intermediate 2	Classical, POST	Par	Tth	2.8
14	Svidritskiy, E, et al. (2014)[12]	3J78*	16.7	38.9	0.8	2.8	P, E	Intermediate 2	Classical, POST	-	Sce	6.3
15	Feng ,S, et al (2013)[13]	4V8U	18.2	40.6	1.0	2.8	P, E	Intermediate 2	Classical, POST	EF-G.GDP	Tth	3.7
16	Gao, YG, et al. (2009)[14]	4V5F	18.5	40.5	0.8	3.1	P, E	Intermediate 2	Classical, POST	EF-G-GDP, Fus	Tth	3.6
17	Zhou, J, et al. (2014)[15]	4W29	18.5	40.1	3.3	21.9	ap/ap, pe/E	Intermediate 1	Chimeric hybrid	EF-G.GDP, Fus	Tth	3.8
18	Ramrath, D, et al. (2013)[16]	4V7B*	19.1	38.0	3.4	19.3	ap/P, pe/E	Intermediate 1	Chimeric hybrid	EF-G.GDP, Fus	Eco	6.8
19	Ratje, AH , et al. (2010)[17]	4V5N*	20.4	42.2	3.9	20.4	pe/E	Intermediate 1	Chimeric hybrid	EF-G.GDP, Fus	Tth	7.6
20	Zhou, J, et	4V9J	20.6	41.5	4.2	21.3	pe/E	Intermediate 1	Chimeric	EF-	Tth	3.9

	al. (2013)[18]								hybrid	G.GDPNP, Vio		
21	Zhou, J, et al. (2013)[18]	4V9L	21.5	43.1	2	18.5	pe/E	Intermediate 1	Chimeric hybrid	EF-G.GDP, Fus, Vio	Tth	3.5
22	Zhou, J, et al. (2013)[18]	4V9M	21.5	43	1.9	18.6	pe/E	Intermediate 1	Chimeric hybrid	EF-G.GDP, Fus	Tth	4.0
23	Zhou, J, et al. (2013)[18]	4V9K	21.8	43.1	2.1	18.2	pe/E	Intermediate 1	Chimeric hybrid	EF-G.GDPNP, Vio	Tth	3.5
24	Voorhees R.M., et al. (2014)[19]	3J7R*	21.9	46.1	9.0	5.0	A/P, P/E	Closed	Hybrid	Sec-61	Ssc	3.9
25	Sun, M., et al. (2015)[3]	3JBO*	24.7	-	10.4	3.6	P/E	Closed	Hybrid	-	Pfa	5.8
26	Svidritskiy, E, et al. (2014)[12]	3J77*	25.6	46.2	10.9	5.5	P/E	Closed	Hybrid	-	Sce	6.2
27	Zhang, J, et al.(2016)[20, 21]	3JBV*	26.1	44.3	9.3	6	P/E	Closed	Hybrid	SecM	Eco	3.3
28	Brilot, AF, et al. (2013)[21]	4V7D*	27.1	47.5	10.6	5.9	A/P, P/E	Closed	Hybrid	EF-G.GDP, Vio	Eco	7.6
29	Ratje, AH, et al. (2010)[17]	4V5M*	27.5	50.5	7.4	6.1	P/E	Closed	Hybrid	EF-G.GDP, Fus	Tth	7.6
30	Dunkle, J., et al. (2011)[1]	4GD1	28.3	48.4	9.2	4.8	P/E	Closed	Hybrid	RRF	Eco	3.0
31	Tourigny, DS, et al (2013)[22]	4V9H	28.6	62.5	7.6	6.5	P/E	Closed	Hybrid	EF-G.GDPCP	Tth	2.9
32	Chen, Y., et al. (2013)[23]	4V90	28.7	63	7.4	6.1	P/E	Closed	Hybrid	EF-G.GDPCP, Fus	Tth	3.0

Footnotes:

[a] PDB IDs for X-ray and cryo-EM (denoted with an asterisk) derived ribosome structures; Structures of classical and hybrid state ribosomes, with open and closed L1 stalks (PDB ID: 4GD1 and 4GD2) from Dunkle et al. (2011)[1] are now combined as 4V9D in the PDB database. The open position of the L1 stalk from Dunkle et al. (2011)[1] (PDB ID: 4GD2) was used as the reference state. PDB IDs 4WOI and 5F8K represent multiple ribosome structures; among these, the classical state ribosome with a vacant E site was included in the analysis here.

[b] Magnitude of rotation of the L1 stalk with respect to the L1 stalk open position in PDB ID 4GD2, calculated using the Euler - Rodrigues (E-R) formula, as described in the Methods.

[c] L1 stalk displacement calculated between the positions of P2127 (or 2469 in eukaryotic structures) at the apex of the L1 stalk head domain in each structure with respect to its position in the reference structure, 4GD2. L1 stalk head domain was not modeled in the Plasmodium ribosomes (3JBO, 3JBP, 3JBN) and hence the distances of L1 stalk movement in these structures have not been calculated.

[d] Intersubunit (or Body Rotation), calculated using the E-R method between 16S body domains, with respect to 4GD2 as the reference state[24].

[e] Head Rotation, calculated using the E-R method, using Selmer et al. (2006)[11] (PDB ID: 4V51, formerly, 2J00) as the reference state, as described in Mohan et al 2014[24].

[f] Classification of L1 stalk conformation as Open, Closed, intermediate 1, intermediate 2, corresponding with the functional states of the ribosome.

[g] Classification of ribosome functional states based on the combination of HR, BR and the tRNA binding state.

[h] Peptides and antibiotics cocrystalized with the ribosome. SecM – secretion monitor protein, Bac7 – bactenecin-7, EF-G – Elongation Factor G bound with GDP (guanosine diphosphate), or non-hydrolyzable analogs of guanosine triphospate (GDPNP or GDPCP), EF4 – elongation factor 4, Par – paromomycin, Vio – viomycin, Fus – fusidic acid.

[i] Species of the ribosome. Eco – *Escherichia coli*, Tth – *Thermus thermophilus*, Sce – *Saccharomyces cereviceae*, Pfa – *Plasmodium falciparum,* Ssc – *Sus scrofa*, Hsa – *Homo sapiens,* Yps - *Yersinia pseudotuberculosis.* Eukaryotic structures listed here were not included in the axis deviation analysis reported in Figure 3A. However, we observe that the L1 stalk conformations in these structures occupy similar conformational states as the bacterial structures. The hinge points coincide with those observed in bacterial structures.

[j] Resolution of the structure, as reported in the PDB database. Ribosome structures reported with resolution of 8Å or better for cryo-EM data, and 4Å or better for X-ray crystallography data were included in this analysis.

[*] Cryo-EM derived structures.

Supplementary Figure 1

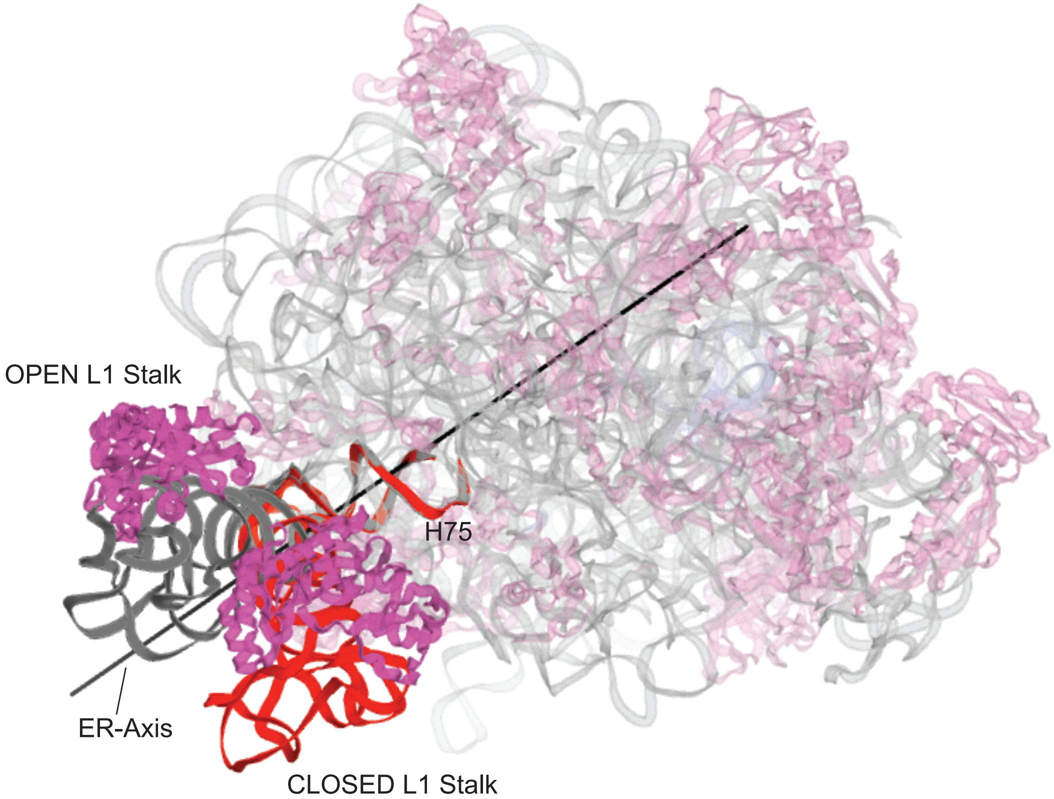

Supplementary Figure 1. Rotation of the L1 stalk from the open to closed states. The Euler – Rodrigues (E-R) axis of rotation (black) of the L1 stalk lies at the base of the L1 stalk and is roughly parallel to helix 75. The E-R transform treats the movement of the L1 stalk as a rigid-body rotation about a single calculated axis (see Methods). The open state of the L1 stalk (dark grey) is from a crystal structure of a classical-state ribosome (PDB ID: 4GD2[1]). The closed state of the L1 stalk (red) is from the structure of a hybrid-state ribosome (PDB ID:4V9D[22]). Protein L1 (magenta) was not modeled in the open state structure (PDB entry 4GD2), but docked from 4V51[11].

Supplementary Figure 2

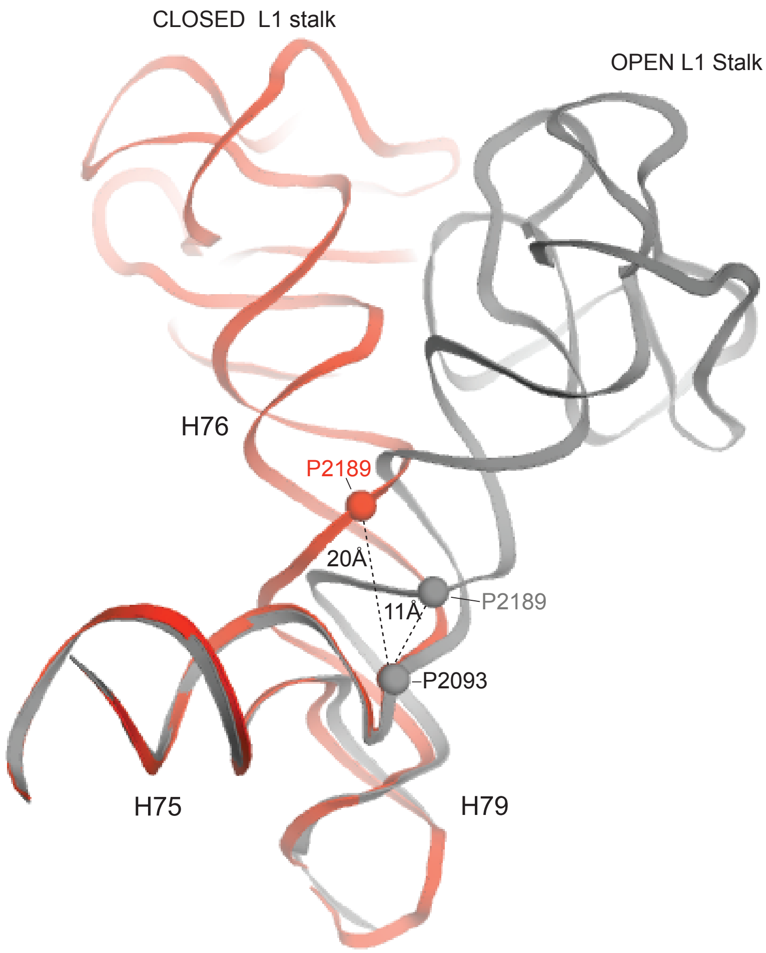

Supplementary Figure 2. Widening of the major groove at the base of H76 during rotation of the L1 stalk. The major groove widens from 11Å in the open (grey) state (PDB ID: 4GD2[1]) to 20Å in the closed (red) position (PDB ID: 4V9H[19]), as measured between the phosphorus atoms at positions 2093 and 2189 (shown as spheres).

Supplementary Figure 3

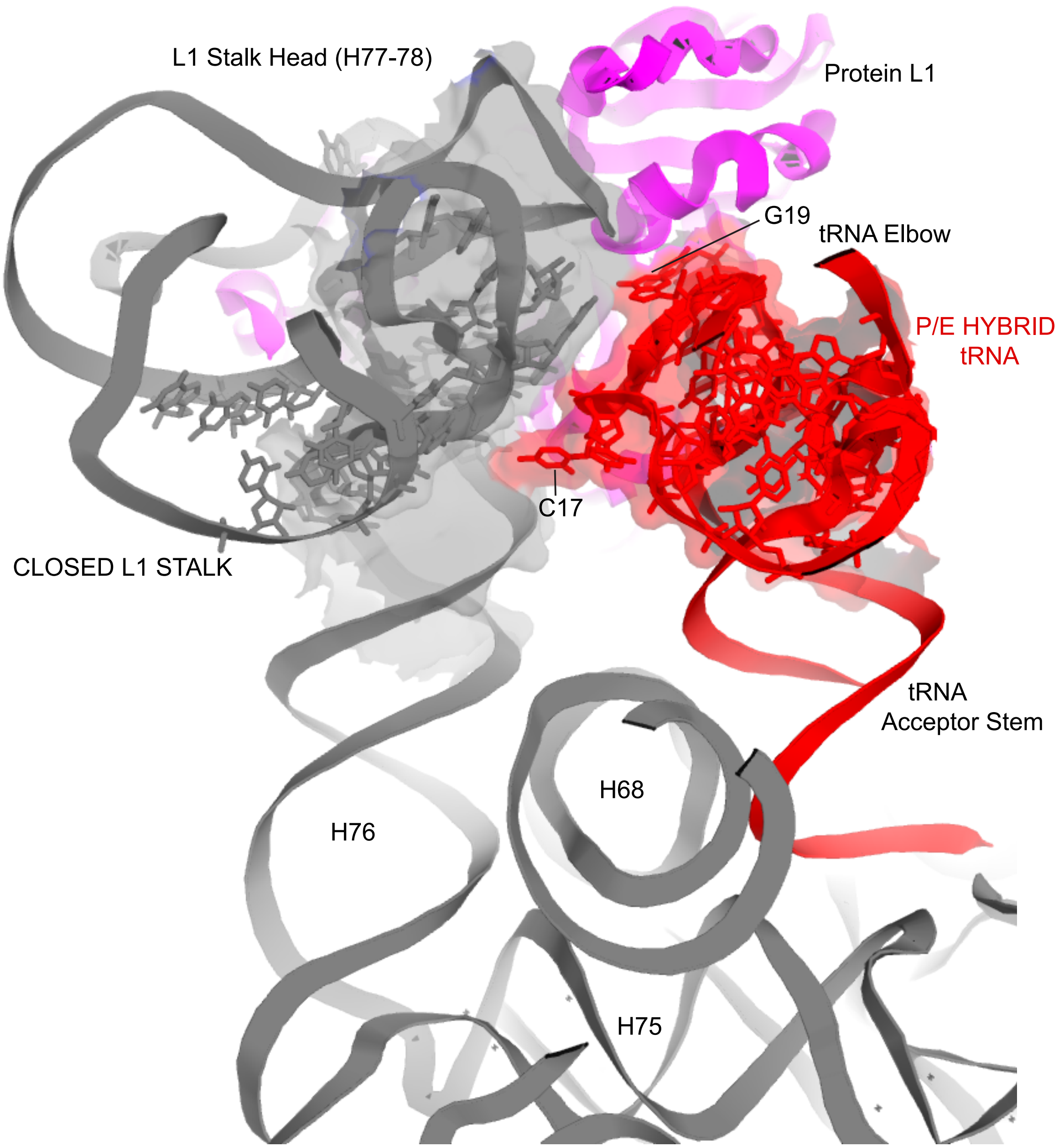

Supplementary Figure 3. Contact between the head of the L1 stalk and the elbow of the tRNA. Besides stacking between the tertiary Watson-Crick pair G19-C56 at the surface of the tRNA elbow and the non-canonical G2112-A2169 purine-purine pair in 23S rRNA moiety of the head of the L1 stalk, the elbow of the P/E tRNA docks on the complementary surface formed by the junction between the head and stalk domains of the closed L1 stalk[23]. Components are protein L1 (magenta); 23S rRNA (grey), P/E hybrid-state tRNA (red). (Magenta – protein L1, grey – 23S rRNA, red – P/E hybrid tRNA). PDB ID: 4V9H[23].

Supplementary Figure 4

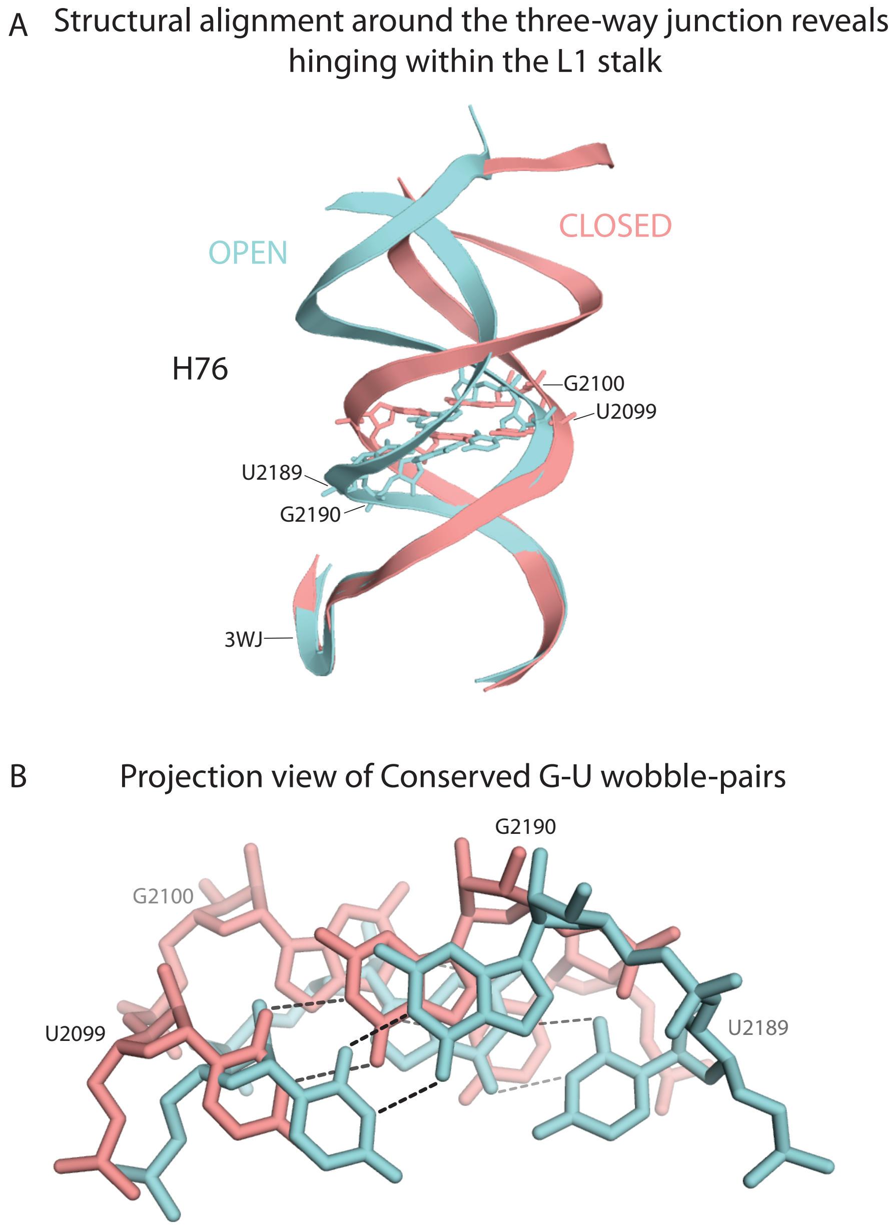

Supplementary Figure 4: Superimposition of the static end helix H76 beyond the three-way junction reveals the hinge point around the G-U rich region of the L1 stalk. The G-U pairs are displaced towards the minor groove of the helix as the stalk moves from the open (cyan, PDB ID: 4GD2[1]) to closed (pink, PDB ID: 3JBV[10]) states. The movement is accompanied by differences in atomic positions of the backbone preceding the G-U region, prominently around positions 2098, 2099 and 2190. This movement results in the widening of the major groove of H76 as shown in figure S2. Contact between G2100-U2089 and H68 is established in the P/E hybrid state, in bacterial as well as eukaryotic L1 stalks in the closed positions.

Supplementary Figure 5

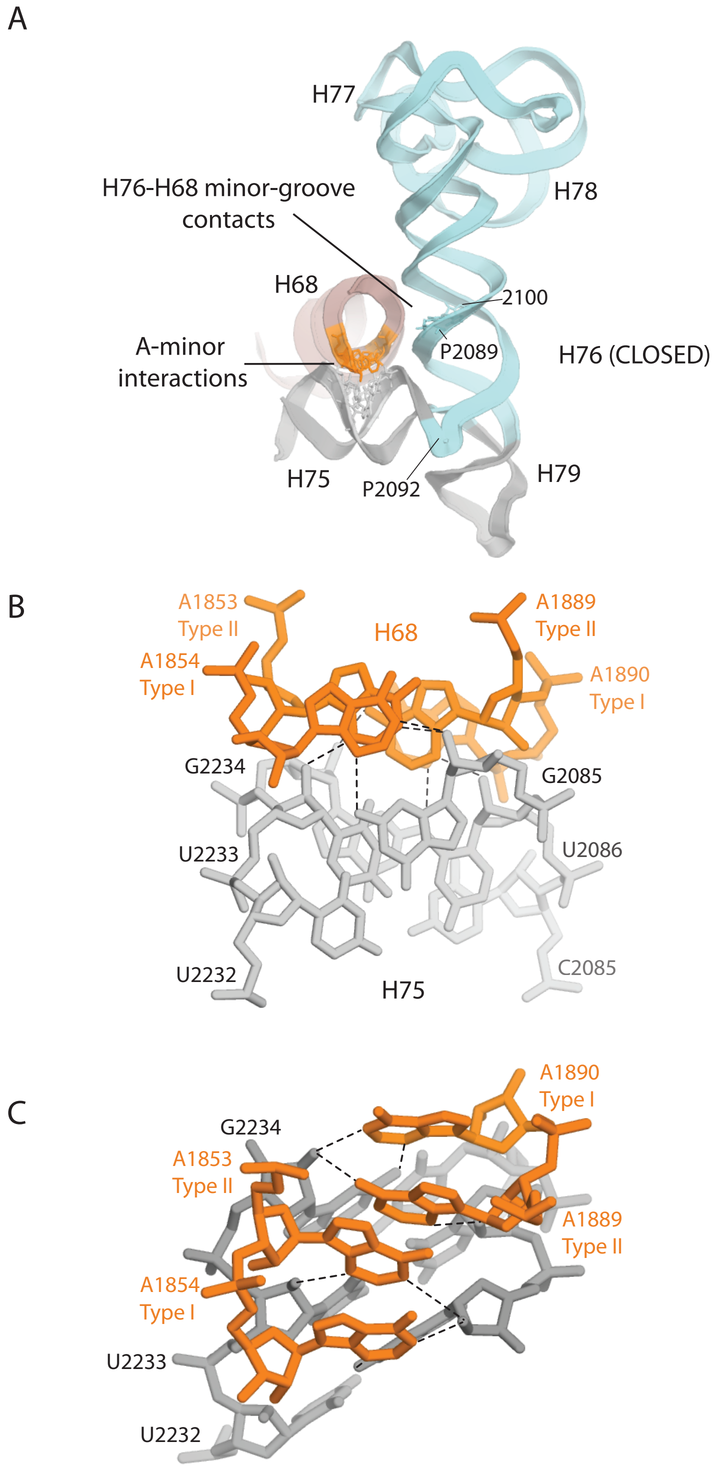

Supplementary Figure 5. A-minor interactions between helices H68 and H75 are maintained throughout the movements of the L1 stalk. These interactions are alternating Type I and Type II A-minor contacts[25] made by four consecutive adenosine

residues in the minor groove of H68 to the minor groove of H75 (also observed in the eukaryotic structures examined) . (A) The A-minor interactions are positioned within the minor groove adjacent to the contact between H76 of the L1 stalk and H68. (B) Molecular interactions between H75 and H68 at the minor groove interface. (C) View of the A-minor interactions from H68, perpendicular to the view shown in panel (B). Shown here, P/E hybrid state, PDB ID: 4V9H[22].

Supplementary Figure 6

a L1 Stalk OPEN

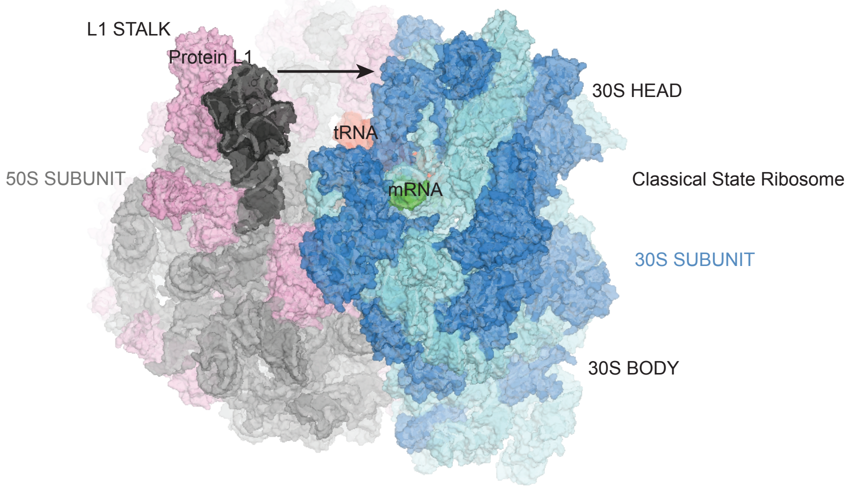

b L1 Stalk CLOSED

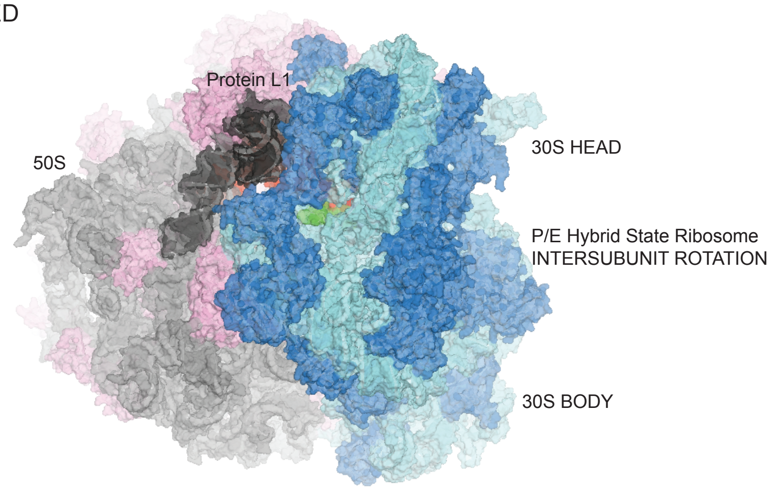

c

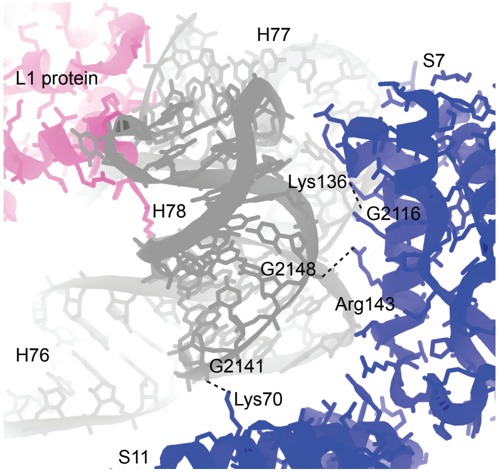

Supplementary Figure 6. Contact between the head of the L1 stalk and the 30S subunit. The L1 stalk moves from the (A) open (PDB ID:4GD2[1]) to a (B) closed ((PDB ID:4V9D[22]) position, to contact the 30S subunit, correlated with translocation of the tRNA from the classical P/P to the hybrid P/E binding state. The ribosome undergoes a large-scale intersubunit rotation, which appears to be coordinated with rotation of the L1 stalk. Components are 50S subunit proteins (pink), 23S rRNA (grey), L1 stalk (dark grey); 16S rRNA (cyan), 30S subunit proteins (blue), mRNA (green), tRNA(red). (C) Molecular interactions between the head of the L1 stalk and the 30S subunit at proteins S7 and S11.

Supplementary Fig 7

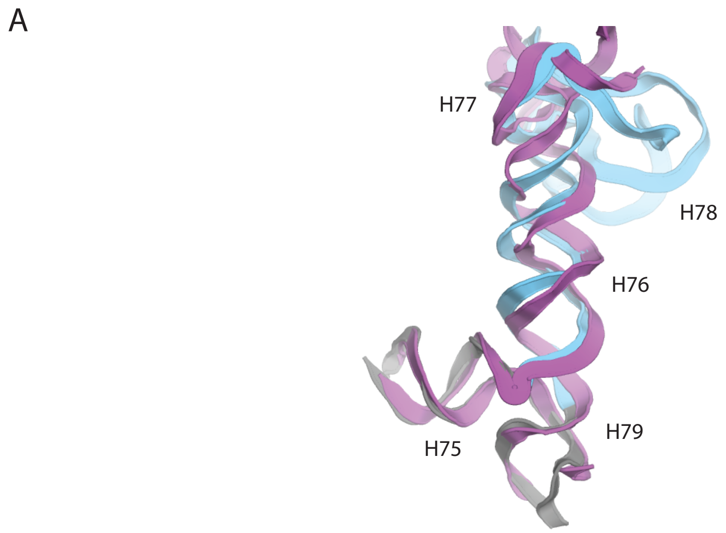

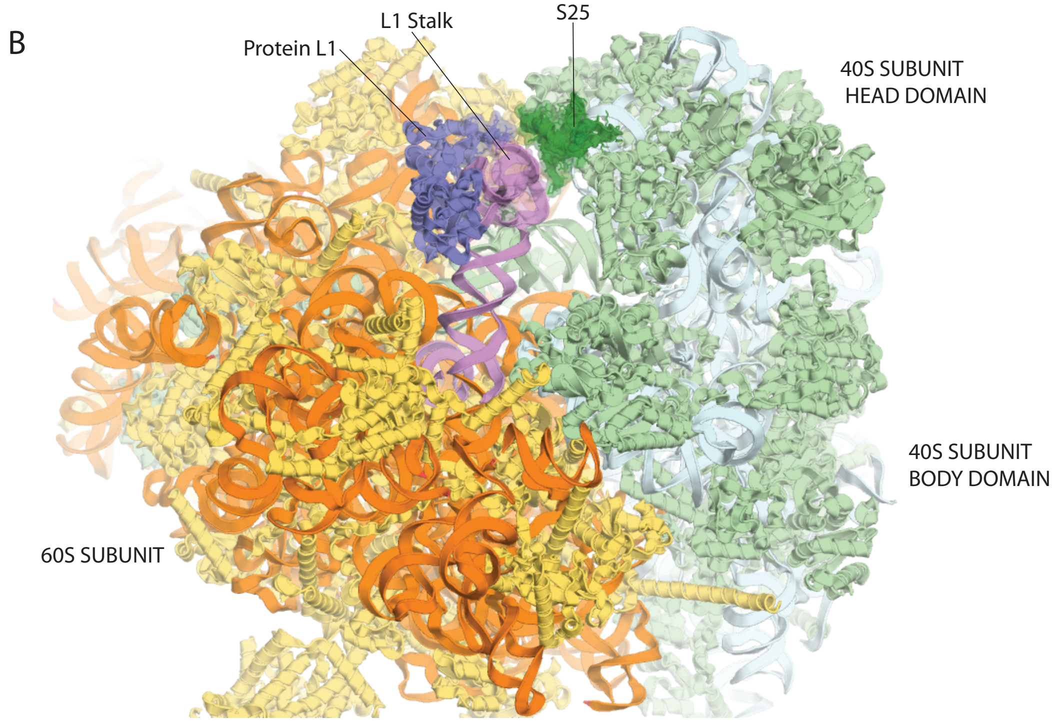

Supplementary Figure 7. The eukaryotic L1 stalk. (A) Superimposition of eukaryotic and bacterial L1 stalks shows their structural similarity. The lengths of the stalks, positions of the three-way junctions and the topology of the helices around the junction are identical (yeast[12] and mammalian[5,6,19]); this may be replaced by disordered insertions[5]. The bacterial helix H78 is absent in most eukaryotic structures. (B) In spite of

the lack of helix H78, in its closed position, the head of the L1 stalk contacts the 40S subunit at protein S25. In this structure (PDB ID: 3J7R[19]), currently the sole representative of a fully modeled L1 stalk in the P/E hybrid state, no contact is seen between the head of the L1 stalk and body of the 40S subunit, unlike that observed in bacteria.

Supplementary Figure 8

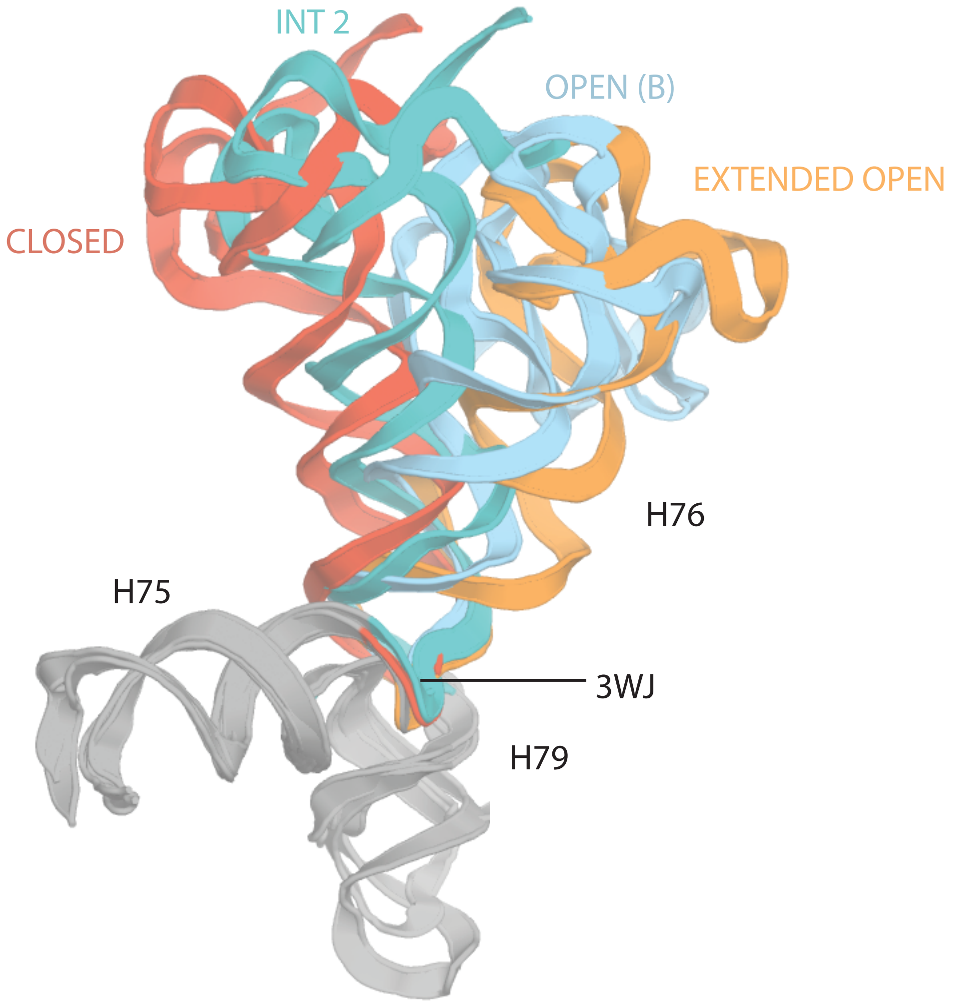

Supplementary Figure 8. Movement of the eukaryotic L1 stalk resembles that of bacterial ribosomes. The majority of the movement originates at the base of the stalk, at the three-way junction between the eukaryotic equivalents of helices H75, 76 and H79. The extended open position is observed in the presence of IRES elements. (PDB IDs: Open (reference) – bacterial 4GD2[1], Closed – 3J7R[19], Intermediate 2 – 5AJ0[6], Extended open – 5IT7[26].

SUPPLEMENTARY REFERENCES

1 Dunkle, J. A. *et al.* Structures of the Bacterial Ribosome in Classical and Hybrid States of tRNA Binding. *Science* **332**, 981-984, doi:10.1126/science.1202692 (2011).

2 Wasserman, M. R. *et al.* Chemically related 4,5-linked aminoglycoside antibiotics drive subunit rotation in opposite directions. *Nat Commun* **6**, doi:10.1038/ncomms8896 (2015).

3 Sun, M. *et al.* Dynamical features of the Plasmodium falciparum ribosome during translation. *Nucleic Acids Res.* **43**, 10515-10524, doi:10.1093/nar/gkv991 (2015).

4 Seefeldt, A. C. *et al.* Structure of the mammalian antimicrobial peptide Bac7(1-16) bound within the exit tunnel of a bacterial ribosome. LID - gkv1545 [pii].

5 Khatter, H., Myasnikov, A. G., Natchiar, S. K. & Klaholz, B. P. Structure of the human 80S ribosome. *Nature* **520**, 640-645, doi:10.1038/nature14427 (2015).

6 Behrmann, E. *et al.* Structural Snapshots of Actively Translating Human Ribosomes. *Cell* **161**, 845-857, doi:http://dx.doi.org/10.1016/j.cell.2015.03.052 (2015).

7 Jenner, L., Demeshkina, N., Yusupova, G. & Yusupov, M. Structural rearrangements of the ribosome at the tRNA proofreading step. *Nat Struct Mol Biol* **17**, 1072-1078 (2010).

8 Korostelev, A. *et al.* Crystal structure of a translation termination complex formed with release factor RF2. *Proc. Natl. Acad. Sci. U. S. A.* **105**, 19684-19689, doi:10.1073/pnas.0810953105 (2008).

9 Li, W. *et al.* Activation of GTP hydrolysis in mRNA-tRNA translocation by elongation factor G. LID - e1500169 [pii].

10 Zhang, D. *et al.* EF4 disengages the peptidyl-tRNA CCA end and facilitates back-translocation on the 70S ribosome. *Nat Struct Mol Biol* **23**, 125-131, doi:10.1038/nsmb.3160 (2016).

11 Selmer, M. *et al.* Structure of the 70S ribosome complexed with mRNA and tRNA. *Science* **313**, 1935-1942 (2006).

12 Svidritskiy, E., Brilot, A. F., Koh, C. S., Grigorieff, N. & Korostelev, A. A. Structures of yeast 80S ribosome-tRNA complexes in the rotated and non-rotated conformations. *Structure (London, England : 1993)* **22**, 1210-1218, doi:10.1016/j.str.2014.06.003 (2014).

13 Feng, S., Chen, Y. & Gao, Y. G. Crystal structure of 70S ribosome with both cognate tRNAs in the E and P sites representing an authentic elongation complex. *PLoS ONE* **8**, 19 (2013).

14 Gao, Y.-G. *et al.* The Structure of the Ribosome with Elongation Factor G Trapped in the Posttranslocational State. *Science* **326**, 694-699, doi:10.1126/science.1179709 (2009).

15 Zhou, J., Lancaster, L., Donohue, J. P. & Noller, H. F. How the ribosome hands the A-site tRNA to the P site during EF-G‚Äìcatalyzed translocation. *Science* **345**, 1188-1191, doi:10.1126/science.1255030 (2014).
16 Ramrath, D. J. F. *et al.* Visualization of two transfer RNAs trapped in transit during elongation factor G-mediated translocation. *Proc. Natl. Acad. Sci. U. S. A.*, doi:10.1073/pnas.1320387110 (2013).
17 Ratje, A. H. *et al.* Head swivel on the ribosome facilitates translocation by means of intra-subunit tRNA hybrid sites. *Nature* **468**, 713-716 (2010).
18 Zhou, J., Lancaster, L., Donohue, J. P. & Noller, H. F. Crystal structures of EF-G-ribosome complexes trapped in intermediate states of translocation. *Science* **340**, 1236086 (2013).
19 Voorhees, Rebecca M., Fernández, Israel S., Scheres, Sjors H. W. & Hegde, Ramanujan S. Structure of the Mammalian Ribosome-Sec61 Complex to 3.4 Å Resolution. *Cell* **157**, 1632-1643, doi:http://dx.doi.org/10.1016/j.cell.2014.05.024 (2014).
20 Zhang, J. *et al.* Mechanisms of ribosome stalling by SecM at multiple elongation steps. LID - 10.7554/eLife.09684 [doi] LID - e09684 [pii].
21 Brilot, A. F., Korostelev, A. A., Ermolenko, D. N. & Grigorieff, N. Structure of the ribosome with elongation factor G trapped in the pretranslocation state. *Proc. Natl. Acad. Sci. U. S. A.* **110**, 20994-20999, doi:10.1073/pnas.1311423110 (2013).
22 Tourigny, D. S., Fernandez, I. S., Kelley, A. C. & Ramakrishnan, V. Elongation factor G bound to the ribosome in an intermediate state of translocation. *Science* **340**, 1235490 (2013).
23 Chen, Y., Feng, S., Kumar, V., Ero, R. & Gao, Y.-G. Structure of EF-G - ribosome complex in a pretranslocation state. **20**, 1077-1084 (2013).
24 Mohan, S., Donohue, J. P. & Noller, H. F. Molecular mechanics of 30S subunit head rotation. *Proc. Natl. Acad. Sci. U. S. A.* **111**, 13325-13330, doi:10.1073/pnas.1413731111 (2014).
25 Nissen, P., Ippolito, J. A., Ban, N., Moore, P. B. & Steitz, T. A. RNA tertiary interactions in the large ribosomal subunit: The A-minor motif. *Proc. Natl. Acad. Sci. U. S. A.* **98**, 4899-4903, doi:10.1073/pnas.081082398 (2001).
26 Murray, J. *et al.* Structural characterization of ribosome recruitment and translocation by type IV IRES. *eLife* **5**, e13567, doi:10.7554/eLife.13567 (2016).
27 Sussman, J. L. *et al.* Protein Data Bank (PDB): Database of three-dimensional structural information of biological macromolecules. *Acta Crystallogr., Sect D: Biol. Crystallogr.* **54**, 1078-1084 (1998).
28 The PyMOL Molecular Graphics System (DeLano Scientific LLC, San Carlos, CA, USA).
29 Lavery, R., Moakher, M., Maddocks, J. H., Petkeviciute, D. & Zakrzewska, K. Conformational analysis of nucleic acids revisited: Curves+. *Nucleic Acids Res.* **37**, 5917-5929, doi:10.1093/nar/gkp608 (2009).
30 Version 7.13 (R2011b) (The Mathworks Inc., 2011).
